# Relationships of Balance, Gait Performance, and Functional Outcome in Chronic Stroke Patients: A Comparison of Left and Right Lesions

**DOI:** 10.1155/2015/716042

**Published:** 2015-10-25

**Authors:** Priscila Garcia Lopes, José Augusto Fernandes Lopes, Christina Moran Brito, Fábio Marcon Alfieri, Linamara Rizzo Battistella

**Affiliations:** ^1^Clinical Research Center, Institute of Physical Medicine and Rehabilitation, University of São Paulo School of Medicine, Rua Domingo de Soto 100, Vila Mariana, 04116-030 São Paulo, SP, Brazil; ^2^Health Promotion and Physical Therapy Faculty, São Paulo Adventist University Center, São Paulo, Brazil; ^3^School of Medicine, University of São Paulo, São Paulo, Brazil

## Abstract

*Introduction*. This study compared the balance by center of pressure (COP) and its relationship with gait parameters and functional independence in left (LH) and right (RH) chronic stroke patients. *Methods*. In this cross-sectional study, twenty-one hemiparetic stroke patients were assessed for Functional Independence Measure (FIM), balance with a force platform, and gait in the Motion Analysis Laboratory. *Results*. The amplitudes of the COP in the anteroposterior and mediolateral directions were similar in both groups. The anteroposterior direction was greater than the mediolateral direction. Only the temporal parameters showed any statistically significant differences. The LH showed a significant correlation between stride length, step length, and gait velocity with COP velocity sway for the healthy and paretic lower limbs. In both groups, the area of COP was significantly correlated with stride length. Motor FIM was significantly correlated with the COP in the LH group. *Conclusion*. There was no difference in the performance of balance, gait, and functional independence between groups. The correlation of the COP sway area with stride length in both groups can serve as a guideline in the rehabilitation of these patients where training the static balance may reflect the improvement of the stride length.

## 1. Introduction

Stroke is the leading cause of disability in adults. Forty percent of stroke patients exhibit moderate functional impairment, and 15% to 30% exhibit severe disabilities [1]. Although intensive rehabilitation, evaluation of the balance, gait, and functional independence are offered to many patients within six months of a stroke, many of them continue to have motor deficits [2]. Adequate therapy increases chronic patient survival, [3] emphasizing the importance of evaluating the overall motor profile following the initial recovery period. Therefore, research in the chronic phase of the stroke is also important.

Postural instability is a common finding and is cited as the leading cause of falls and limited functional independence in stroke patients [4–6]. Posture or balance deficits are common mainly because the unaffected limb bears a greater proportion of the body weight [6–10]. In hemiparetic patients, postural oscillation while standing upright is characterized by an asymmetric profile with larger oscillations on the paretic side than the nonparetic side and low temporal synchronization between oscillations of the lower limbs and the pelvis and between the lower limbs. Difficulty in stabilizing the pelvis and the distal segments of the lower limb on the affected side are reflected in the increase in postural oscillation of hemiparetic patients [11].

Evidence of the differences between the functional consequences of strokes in the left and right hemispheres is particularly interesting. The left hemisphere is more important for motor control, while the right hemisphere is more important for spatial orientation [12]. Motor activities requiring planning and coordination are more dependent on the left hemisphere and are strongly affected in individuals with right side hemiparesis [13, 14]. Right hemisphere lesions are more likely to result in deficits in attention and contralateral perception [15] and stabilization of the position in relation to lesion of left side [14]. The right hemisphere integrates sensorimotor information which is critical for maintaining posture and maintaining sitting or standing positions [15].

Left hemiparetic patients exhibit poorer postural balance in sitting and standing positions compared to right hemiparetic patients, and patients who have not received adequate therapy exhibit high degrees of postural alterations [4]. Some studies have assessed balance by analyzing postural instability during standing in hemiparetic stroke patients [5, 6, 8, 9, 16–19]. These studies were performed mainly in the first year after stroke. Postural instability is assessed through center of pressure (COP) sway analysis, and COP position can be assessed directly using force platforms during the evaluation of posture and gait.

Some studies have reported higher anterior-posterior oscillation than mediolateral oscillation in static COP assessments [20–22]. Rode et al. [8] compared postural oscillation in 15 right and 15 left hemiparetic patients and found that the latter group exhibited larger areas of oscillation and mediolateral displacement. Other studies have attempted to correlate static balance data with gait parameters [20, 23].

The correlations of balance and gait parameters are important for the assessment and rehabilitation of patients because a reliable correlation could mean that resources used to improve balance could also influence gait. In clinical practice, it is clear that delay in therapy leads to poorer postural control among the left hemiparetic patients, but after one year has passed and rehabilitation is finished, monitoring and comparing with right side hemiparetics is difficult. It would be interesting to discover the possible mechanisms involved in the control of posture, the regulation of skeletal muscle during gait, and the oscillations of the COP that maintain corporal stability in hemiparetic patients. As the stroke sufferer's balance is impaired and can lead to consequences such as falls, knowing the questions related to balance and gait will be important in order for these two physical capabilities to be better understood in hemiparetic subjects. It is also believed that this will lead to a better direction regarding the rehabilitation of these patients.

We hypothesized that the left hemiparetic patients still have poorer balance in the chronic phase as well as in early stage [4]. Therefore the purpose of this study was to compare the balance by COP sway and its relationship with gait parameters and functional independence in left or right chronic stroke patients.

## 2. Methods

This cross-sectional study was conducted at the Institute of Physical Medicine and Rehabilitation at the Clinical Hospital of the Medical School of the University of São Paulo (IMREA-HCFMUSP). Twenty-seven hemiparetic chronic stroke patients treated at the hemiplegia outpatient clinic were invited to participate in the study. Selected patients were informed of the study's aims and procedures, and they or their caretakers signed an informed consent form. This research project was approved by the Committee on Ethics and Research (CAPPesq) under protocol number 0280/09.

The following inclusion criteria were used: hemiparesis resulting from a stroke that occurred at least 12 months prior to the study; age between 45 and 65 years; pattern of hemiparesis featuring brachial predominance; ability to walk 10 meters unassisted; ability to remain standing upright for 60 seconds unassisted; and right side dominance (right-handed individuals). Exclusion criteria were as follows: cognitive impairments affecting comprehension and disabilities arising from other conditions, such as deformity or pain.

### 2.1. Clinical Assessment

Patient clinical records containing personal data, clinical diagnosis, time of the lesion, and Functional Independence Measure FIM [24] score were evaluated. The FIM quantitatively evaluates the care demanded by a person to perform a series of motor and cognitive tasks of daily living. Among the activities evaluated are self-care, transfers, locomotion, sphincter control, communication, and social cognition—including memory, social interaction, and problem solving. Each of these activities is evaluated and receives a score ranging from one (total dependence) to seven (complete independence) and the total score ranges from 18 to 126. Two FIM areas describe the motor score ranging from 13 to 91 points and cognitive score ranging from 5 to 35 points [24].

First, the patient's right or left dominance was assessed by asking about the dominant upper limb (writing hand). The physical assessment involved tests of muscle strength and tone. Muscle strength was tested according to the Kendall scale [25] and spasticity was tested via the modified Ashworth scale [26, 27]. Quadriceps strength was chosen as the representative assessment for the lower limb, and brachial biceps strength was chosen for the upper limb. Spasticity was measured in the gastrocnemius muscle because this muscle is important for the ankle strategy in maintaining postural control [21].

### 2.2. Postural Control Assessment

The postural control assessment was performed with patients standing upright. Patients remained standing with their arms hanging alongside their body, eyes fixed on a point on the wall, and feet set on a force platform (AMTI OR6-7 version 2.0/2004, installed at the Motion Analysis Laboratory). McIlroy and Maki [28] showed that foot separation in a preferred stance position is correlated to subject height and is considerably larger than that usually standardized for posturography suggesting a standard position with 17 cm separation or 11 percent of subject height would avoid uncomfortable foot positions. To accommodate the increased instability expected in chronic stroke patients while still taking anthropometric variations into consideration a foot separation equal to the length of the patient's feet was adopted as measured between the midpoints of both heels. The feet were positioned parallel to each other on the platform. After calibration, COP position was recorded by the force platform.

Patients were asked to hold the position for 60 seconds, and the assessment was repeated three times. The data were acquired at 100 Hz and subsampled at 10 Hz as described by Raymakers et al. [29]. To avoid disturbances from the initial stabilization of the subject, the first 10 seconds of each record were discarded. The center of pressure (COP) sway variables measured were anterior-posterior and mediolateral oscillation amplitude, COP sway area, and average velocity [29]. Sway amplitude was calculated as the difference between the maximum and minimum coordinates of the COP in each direction and was expressed in centimeters (cm); the rectangular area that covered the whole COP trajectory was calculated by multiplying anterior-posterior and mediolateral amplitudes and was expressed in cm^2^. Average sway velocity was calculated by dividing the total length of the COP trajectory by the duration of the recording and expressed in centimeters per second (cm/s). A therapist remained by the patient's side during the procedures for safety in case the patient lost his or her balance. Posturography (postural control) data were recorded by the EVaRT 5.0 software (Motion Analysis Corporation) and we used a routine developed and processed in the Matlab 2008a software (Mathworks) to process the data.

### 2.3. Gait Assessment

To obtain spatial and temporal gait parameters, reflective markers were placed on the patient's heels on the lateral and medial malleoli between the first and second metatarsals and on the sacrum. Considering that joint angles would not be analyzed, this is the minimum subset of the modified Helen Hayes market set [30] that allows the calculation of the desired gait parameters using specialized computer software (Orthotrak 6.2, Motion Analysis Corporation, Santa Rosa, CA, USA). Patients were then asked to walk along a preestablished path measuring 10 meters in length in the Motion Analysis Laboratory. The gait parameters considered were step and stride lengths, gait velocity, and gait cadence. Step and stride lengths were expressed in centimeters, velocity in centimeters per second, and cadence as the number of steps per minute. Temporal parameters considered as percentages of the gait cycle time were also measured: stance phase is the whole period of time when the foot is in contact with the ground; swing phase is the period when the foot is not in contact with the ground; double stance onset is the period when both feet are in contact with the floor at the beginning of the gait cycle; single stance is defined as the percentage of time of the gait cycle when only one foot is in contact with the ground. The data from the markers were captured by eight Hawk System digital cameras operating at 100 Hz using software provided by their manufacturer (EVaRT 5.0, Motion Analysis Corporation, Santa Rosa, CA).

### 2.4. Data Analysis

All data were distributed normally, according to the Kolmogorov-Smirnov test, with *p* < 0.05. Parametric tests, such as Student's *t*-test, were used to compare means to analyze static postural control. The level of significance was set at *p* ≤ 0.05. The COP velocity and area of oscillation for left and right hemiparesis groups were compared. Gait data were also compared between groups using Student's *t*-test. Finally, possible correlations between the gait parameters and static COP sway, velocity, and area were evaluated using Pearson's correlation coefficient, where 0 to 0.30 was interpreted as a weak correlation, 0.30 to 0.70 as a moderate correlation, and over 0.70 as a strong correlation. The static COP sway measured (mean velocity and area of oscillation) was evaluated for correlation with gait data and with the FIM.

## 3. Results

Of the 27 patients contacted from the IMREA-HCFMUSP, six did not complete evaluations; thus 21 participants remained: nine in the right hemiparetic group (RH) group and 12 in the left hemiparetic group (LH).  Table 1 describes the characteristics regarding mean age, time since injury, Functional Independence Measure (FIM), muscle strength, and spasticity of all patients and of each group. The groups are similar, except for the motor FIM, which had a higher score for the RH.

In assessing the extent of COP sway in the anterior-posterior (RH—3.0 ± 1.4 cm and LH—3.1 ± 1.2) and mediolateral directions (RH—1.7 ± 1.2 and LH—1.5 ± 0.5 cm) and the average of the velocity (RH—1.9 ± 1.1 and LH—1.6 ± 0.8 cm/s) it was found that the values were similar (*p* > 0.05) in the right and left sides.

Between groups, only the temporal parameters showed any statistically significant differences. All patients spent more time in the stance phase for the healthy lower limb; specifically the single stance time was significantly different in both the left (*p* = 0.0004) and the right (*p* = 0.001) hemiparesis groups.

In general, the groups showed that the total stance time was longer for the healthy lower limb than for the affected limb; as for the analyzed gait variables (stance, swing, and single stance), there were statistically significant differences between healthy and affected limbs.

There were no statistically significant differences between the affected and healthy sides in either group of patients for the remaining parameters, such as length of step and stride, gait velocity, initial stance, and cadence. The step length of the affected limb was longer than that of the healthy limb in most of the patients in both groups. Meanwhile, the stride length of the healthy limb was greater than that of the affected limb in both groups.

In both groups the mean velocity and cadence were similar and had low standard deviations; there were no statistically significant differences in these gait parameters between patient groups. The LH had a mean velocity of 40.7 (±3.6) cm/s and a mean cadence of 74 (±0.99) steps/minute; the RH had a mean velocity of 40 (±1.5) cm/s and a cadence of 77.6 (±3.6) steps/minute. Higher values were recorded for the healthy lower limb in most patients (LH, *n* = 6; and RH, *n* = 3).

Correlations between the gait parameters and static COP velocity were also analyzed (Table 2). The left hemiparetic group showed a significant (*p* < 0.05) correlation between stride length, step length, and gait velocity with COP velocity sway for the healthy and paretic lower limbs. In the healthy limb, the parameters like swing and stance were significantly correlated with the COP velocity.

Table 3 shows the correlation coefficients between the area of COP, sway, and gait parameters. In both groups the area of COP was significantly correlated with stride length. In the right group the step length was significantly correlated with the area of COP but only in the healthy limb. By contrast, these variables were moderately correlated in both limbs of left hemiparetic patients. In this group gait velocity and COP area were significantly correlated. The area of COP in the healthy limb was significantly correlated with durations of the stance and swing phases and in the affected limb it was significantly correlated with the single stance duration and double stance onset.

The correlations between the FIM and static balance data were also analyzed. Both groups had their area of COP sway moderately correlated with motor and the FIM total. Motor FIM was significantly correlated with the area of COP sway in the left hemiparesis group. The velocity of COP sway was not significantly correlated with the FIM (Table 4).

## 4. Discussion

The main aim of this study was to compare the balance by COP sway and its relation with gait parameters and functional independence in left and right chronic stroke patients. The results showed no differences in the performance of balance, gait, and functional independence between groups with chronic damage to the right or left hemisphere. The chronicity of the patients in this study may have contributed to higher motor adaptation.

As in the present study, Peurala et al. [31] also found no difference in the COP oscillation velocity in either the sagittal or frontal plane among individuals with chronic hemiparesis. Ioffe et al. [32] conducted a study about learning postural control with two groups of hemiparetic patients. Patients were trained in 10 sessions consisting of 2 activities in which they had to displace COP visualized on a screen. In one postural control learning activity, patients with left hemiparesis exhibited a long delay during the initial sessions, while patients with right hemiparesis learned faster; however, the learning speed was similar in both groups after 10 days. The authors argued that specific control of the COP trajectory may require a large amount of sensory information, which is associated with the right hemisphere.

Many studies have been conducted on patients during the first months after a stroke. The evidence that patients can still learn with time and stimulation raises questions about the evolution of chronic patients who were or are still being subjected to rehabilitation training and about the lateralization of lesion effects. The participants in this study all suffered from lesions more than 12 months before being tested, with an average time of 32 months since lesion. Muscle strength and tone were evaluated to characterize the pattern of injury patients in the area of the middle cerebral artery, which corresponds to a greater involvement of the upper limb in the hemiparesis. The average age of patients (55 years) of this study was not regarded as elderly, and this may not have affected the balance analysis. According to Ruwer et al. [33] aging affects the ability of the central nervous system to process the vestibular, visual, and proprioceptive signals responsible for maintaining body balance and reducing the ability to modify adaptive reflexes. In this study, the mean static COP sway was not statistically significant. When static balance was tested on force platforms, patients in both groups exhibited greater anterior-posterior COP sway than mediolateral one. According to a literature review on falls in stroke patients by Weerdesteyn et al. [34], these patients exhibit greater body oscillation, especially in the frontal plane, and rely more on the healthy limb to maintain balance. Depending on how oscillation is measured, it can be as much as 1.5 to 5 times greater in stroke patients than in healthy individuals.

Despite this result, mediolateral sway continues to be considered as the best prognostic parameter for revealing balance and risk of falling in hemiparetic patients [6, 29, 35]. To Kadaba et al. [30], the anteroposterior displacement of the COP was faster than the medial-lateral one only among patients after a stroke; it did not occur among healthy subjects.

Although the functional independence results did not reveal a significant difference between the groups, according to Peurala et al. [31], we can assume that the loss of the dominant limb has a greater effect on daily activities than loss of the nondominant limb. The right hemiparesis group represents the loss of the dominant limb because all patients were right-handed. Right hemiparetic patients exhibited better functional abilities, especially in activities involving standing upright, balance, and gait. Areas involved in body schema and spatial perception are affected in left hemiparetic patients. From a neurophysiological perspective, this factor contributes negatively to kinesthetic sensation and perception, eventually leading to the neglect of the affected half of the body [8, 10].

The results for temporal gait parameters, such as duration of the stance and swing phases, were consistent with those in the literature [36, 37]. Dynamic analysis revealed that the healthy limb supported the body weight longer in both groups and spent significantly less time in the swing phase compared with the affected limb. This shows that, despite time and rehabilitation, a persistent difference in muscle strength and perception in the affected lower limb affects gait symmetry. The remaining gait parameters assessed, such as step length, stride length, velocity, stance, and cadence, did not differ significantly between healthy and affected limbs. In stroke patients, the lengths of steps and strides are typically different between healthy and affected limbs, and the length of the gait cycle increases, the stance time increases, the duration of the swing phase decreases, the duration of the double stance increases, cadence and velocity decrease [11, 16, 20, 36–40], and stride width increases [41]. The asymmetry in the propulsion of the lower limbs during gait also increases, as reflected by the difference in ground reaction force [42]. However, the groups of patients in this study did not necessarily follow this pattern, likely indicating adaptation and reduction of motor asymmetry over time.

Nardone et al. [20] found a correlation between COP positioning and muscle strength in the nonaffected lower limb during the single stance phase, with COP remaining greater in the healthy limb. There was a positive correlation between single stance duration and COP sway in the healthy lower limb. The asymmetry found in hemiparetic patients affects gait by increasing the time and effort required to shift the body weight to the affected limb. The degree of asymmetry, as measured by stabilometry, was correlated with the level of difficulty reflected in the gait parameters.

Correlations between COP velocity sway, gait parameters, COP area, and motor FIM were greater in the left hemiparesis group. The greater correlation between COP data and gait parameters in patients with right hemisphere lesions might be due to the role of this hemisphere in sensorimotor integration, which is critical for postural maintenance [15]. Right hemisphere lesions have a very large impact and can cause body schema alterations, contralateral neglect, altered postural alignment [13], and visuomotor impairment [9].

The COP area displacement had more positive correlations with gait parameters in both groups relative to the other parameters. This might be due to the mediolateral component of displacement; the area is calculated based on anterior-posterior and mediolateral displacements, and this parameter is significantly different in hemiparetic patients and healthy individuals [8, 16, 20, 32]. Paillex and So [6] observed reductions of lateral COP displacement and the area of COP displacement after rehabilitation in hemiparetic patients. These results were correlated with reduced muscle strength in the thigh adductors and abductors. A quantitative study found correlations among gait performance, postural stability, and functional assessments in hemiparetic patients. Their results showed that the ability to maintain stability while standing and the ability to shift the center of mass were significantly correlated with gait velocity, stride length, and step length in the paretic limb. After correlating the balance scores obtained by the Fugl-Meyer Assessment with gait variables and stability while in a standing position, they concluded that hemiparetic patients compensate for a lack of balance with smaller steps and a slower gait [23]. In the present study, the static balance area was moderately correlated with COP sway and the FIM in both groups; however, static COP velocity was not correlated with the FIM.

We believe that, with time and rehabilitation treatment, a sensorimotor reorganization makes similar performance improvements in balance and gait test regardless of the side lesioned. However, it is believed that rehabilitation programs should be alert to improve balance especially in the anteroposterior direction and include therapeutic exercises that stimulate the different receptors. The COP sway area was correlated with stride length in both groups. This data can serve as a guideline in the rehabilitation of these patients where training the static balance may reflect the improvement of the stride length.

As a limiting factor, we point out the size of our sample, but we believe that the results are not invalidated due to the range of objective assessments carried out. However, we believe that further research should be carried out considering the standardization of brain injury size and the influence of treatment programs on the improvement of postural control and gait parameters associated with the functionality of this population.

## 5. Conclusion

There was no difference in the performance of balance, gait, and functional independence between the groups of chronic hemiparetic stroke patients when comparing left hemisphere lesion and right hemisphere lesion. The chronicity of the patients in this study and rehabilitation treatment may have contributed to higher motor adaptation. The correlation of the COP sway area with stride length in both groups can serve as a guideline in the rehabilitation of these patients where training the static balance may reflect the improvement of the stride length.

Although the group with left hemiparesis has shown better correlation of COP and gait parameters, improved functionality, and gait that are routinely worked in rehabilitation programs, this may be related to the improvement of balance. Yet, it is believed that this should also be emphasized so that these variables are also improved. In particular, the anteroposterior direction should be emphasized in chronic stroke patients.

## Figures and Tables

**Table 1:** Characteristics of the studied patients: motor and total FIM, muscle strength, and spasticity.

	Total (*n* = 21)	RH (*n* = 9)	LH (*n* = 12)	*p*
Male/female	15/6	7/2	8/4	0.65

Mean age (years) (SD)	55.3 (±5.9)	54.2 (±2.8)	56.4 (±7.4)	0.41

Time since lesion (months)	32.6 (±17.7)	37.4 (±19.9)	29 (±15.7)	0.29

BMI (Kg/m^2^)	30.1 (±5.6)	29.5 (±6.8)	30.60 (±5)	0.69

Weight (kg)	73.2 (±10.9)	75.3 (±11.7)	71.4 (±11.6)	0.26

Height (m)	1.5 (±9.36)	1.6 (±9.5)	1.5 (±8.9)	0.20

FIM	Motor	81 (±5.0)	73.7 (±8.7)	0.03
	Total	107 (±16.0)	107 (±8.7)	0.32

Muscle strength	Biceps brachial	3.0 (±0.9)	2.4 (±1.7)	0.4
	Quadriceps	3.8 (±0.4)	3.9 (±0.7)	0.9

Spasticity	Biceps brachial	1.2 (±0.7)	1.0 (±0.7)	0.3
	Gastrocnemius	1.1 (±0.7)	0.8 (±0.7)	0.16

Note: FIM: functional independence measure; RH: right hemiparetic group; LH: left hemiparetic group; SD: standard deviation.

**Table 2:** Correlation between velocity of COP sway and gait parameters.

Gait parameters	LH		RH	
	Healthy limb	Affected limb	Healthy limb	Affected limb
Step length	−0.60^*∗*^	−0.62^*∗*^	0.38	0.36
Stride length	−0.72^*∗*^	−0.72^*∗*^	0.46	0.41
Gait velocity	−0.68^*∗*^	−0.70^*∗*^	−0.05	−0.19
Stance phase	0.76^*∗*^	0.27	0.47	−0.01
Swing phase	−0.76^*∗*^	−0.27	−0.47	0.01
Double stance onset	0.70^*∗*^	0.53	0.07	0.61
Single stance	−0.27	−0.76^*∗*^	0.01	−0.47
Cadence	−0.46	−0.45	−0.58	−0.62

^*∗*^Pearson's correlation coefficient (*r*) with *p* < 0.05; RH: right hemiparetic group; LH: left hemiparetic group.

**Table 3:** Correlation between the area of COP sway and gait parameters.

Gait parameters	LH		RH	
	Healthy limb	Affected limb	Healthy limb	Affected limb
	OE	OE	OE	OE
Step length	−0.48	−0.55	0.74^*∗*^	0.49
Stride length	−0.78^*∗*^	−0.79^*∗*^	0.76^*∗*^	0.71^*∗*^
Gait velocity	−0.71^*∗*^	−0.72^*∗*^	0.31	0.18
Stance phase	0.74^*∗*^	0.27	0.41	0.29
Swing phase	−0.74^*∗*^	−0.27	−0.41	−0.29
Double stance onset	0.38	0.67^*∗*^	0.31	0.59
Single stance	−0.27	−0.74^*∗*^	−0.29	−0.41
Cadence	−0.40	−0.38	0.44	0.49

^*∗*^Pearson's correlation coefficient with *p* < 0.05; R: right hemiparetic group; L: left hemiparetic group.

**Table 4:** Correlations between FIM and balance (COP).

	L Hemiparesis		R Hemiparesis	
	Total FIM	Motor FIM	Total FIM	Motor FIM
Velocity of oscillation	−0.19	−0.28	−0.04	0.12
Area of oscillation	−0.50	−0.59^*∗*^	0.52	0.62

^*∗*^Pearson's correlation coefficient (*r*) with *p* < 0.05.
